# Addressing Lung Cancer Screening Disparities: More Work To Be Done

**DOI:** 10.1016/j.atssr.2024.07.023

**Published:** 2024-08-06

**Authors:** Hollis Hutchings, Olivia Aspiras, Todd Lucas, Ikenna Okereke

**Affiliations:** Department of Surgery, Henry Ford Health, Detroit, Michigan; Charles Stewart Mott Department of Public Health, Michigan State University, Flint, Michigan; Department of Surgery, Henry Ford Health, 2799 W Grand Blvd, Detroit, MI 48202

Reply to the Editor:

We thank Olivera and colleagues^1^ for their thoughtful response to our paper.^2^ As they highlight, racial and gender differences in lung cancer screening continue to encapsulate inequities in lung cancer prevention. In our study, we found that when screening-eligible Black patients were informed of the benefits of lung cancer screening, they were as willing as White patients to obtain this screening. With this knowledge, we are considering how didactic methods can be overlayed with evidence-based messaging strategies to address racial and gender screening disparities. For example, our team has shown that culturally targeted messaging carries a potential to address both racial and gender differences in cancer screening uptake.^3^ We have also shown that medical mistrust is an important factor for some patients.^4^ Ultimately, our goals include identifying discrete ways to inform our patient population better to increase screening uptake. In doing so, we hope to diagnose more early-stage lung cancers for better overall cure rate and survival.

Reducing racial disparities in lung cancer screening must be done utilizing a multitiered approach. There are multiple points in the care of a patient in which social determinants of health negatively affect the likelihood of obtaining screening. Black patients with lung cancer tend to have lower pack-year exposures and be younger in age. When referred, Black patients are less likely to obtain the screening scan and have longer follow-up times.^5^ Black patients are also more likely to be uninsured. To be effective in reducing racial disparities, we must find solutions for each obstacle in the chain of events that leads to relative underscreening of Black patients.

Thank you for your response, and we look forward to conducting further disparity research efforts to improve patient outcomes for society broadly.
